# Influence of Randomness in Rubber Materials Parameters on the Reliability of Rubber O-Ring Seal

**DOI:** 10.3390/ma12091566

**Published:** 2019-05-13

**Authors:** Banglong Liang, Xi Yang, Zili Wang, Xing Su, Baopeng Liao, Yi Ren, Bo Sun

**Affiliations:** School of Reliability and Systems Engineering, Beihang University, Beijing 100191, China; lbl@buaa.edu.cn (B.L.); bryantyx@buaa.edu.cn (X.Y.); 04767@buaa.edu.cn (Z.W.); xs_1818@163.com (X.S.); lbp2016@buaa.edu.cn (B.L.); renyi@buaa.edu.cn (Y.R.)

**Keywords:** rubber O-ring seal, reliability, parameter randomness, influence analysis

## Abstract

The properties of materials directly affect the performance of the sealing structures, among which the rubber O-ring seal is one of the most commonly used. In addition, the performance of the O-ring seal is often influenced by the randomness in structure and working conditions, which greatly reduce the reliability of the sealing structure. This paper proposes a reliability-based method to analyze the influence of the randomness in rubber material parameters on the sealing performance of the O-ring. Based on the failure mechanism of the O-ring seal, the stochastic characteristics of the parameters in O-rings are determined through experiments, and the influences of these parameters on the reliability are subsequently analyzed. Moreover, the working conditions are also taken into account to analyze their influence on the performance and reliability of the O-ring seal. The proposed method provides easy access to estimate the reliability of the rubber O-ring seal considering the uncertainty in structure and operational conditions. It is revealed that the material and geometric parameters had greater influence on the reliability of the rubber O-ring.

## 1. Introduction

Rubber O-ring seals are one of the most commonly used sealing structures in mechanical systems. In sealing structures, failure of the rubber O-ring seal may lead to catastrophic accidents [1,2]. Rubber O-ring seals prevent the leakage of hydraulic oil and help to avoid the scratch caused by the direct contact between the piston and the inner wall of the cylinder block [3]. It is necessary to propose higher requirements for the reliability of the O-ring due to its tough working conditions and the inconvenience of its replacement [4]. In the life cycle of the rubber O-ring seal, various factors may affect its reliability, such as material aging [5,6], ambient conditions [7], and load changes [8]. The randomness of the parameters of these factors will have a certain impact on the reliability of the rubber O-ring seal. Therefore, it is necessary to analyze the randomness of the parameters to discover the influences on the reliability of the rubber O-ring seal, so as to improve the economy and safety of their application.

In recent years, numerous studies focused on the performance of O-rings have been performed. Ruben et al. [9] designed a multifunctional silicone rubber nanocomposite to improve the mechanical properties. The mechanical properties of silicone rubber can also be improved with a ceramifying process [10]. Qiu et al. [11] investigated the impact factors and action mechanism of promoting the thermal oxidative stability of silicone rubber by incorporating reduced graphene oxide. A mechanical model of compression was established to analyze the sealing performance of the rubber [12]. Dara [13] analyzed the radial force produced by the sealing face on the O-ring. Wang et al. [14] studied the sealing characteristics of O-rings under different working conditions. Yang et al. [15] studied the wear and damage behavior of O-rings under harsh conditions. Akulichev et al. [16] studied the effects of different surface treatment processes on sealing performance. In addition, the aging of the rubber significantly affects the physical properties of the seal [17,18]. However, these studies did not carry out reliability-based seal performance analysis [19], which makes it difficult for us to distinguish different reliability requirements under various conditions, and the uncertainties of the working conditions were also ignored.

Based on the analysis of sealing performance, stochastic analysis was introduced to study the influence of one or more stochastic parameters on reliability so that the reliable life of the product can be predicted. The random parameters have obvious effects on the reliability of the products, and the influences were discussed in [20,21]. Various studies have been conducted on the reliability of random systems, such as reliability-based optimization [22,23] and reliability sensitivity analysis [24]. Wen et al. [25] proposed a method of reliability analysis for uncertain random systems based on chance theory. The failure analysis for the rubber belt was conducted using classification models [26]. Marco et al. [27] studied the fatigue life prediction method of materials, but the randomness of parameters was not considered in his study. Woo [28] and Shao [29] predicted the life of sealing structures using the finite element method and physics of failure method, respectively, but the random distribution of only one parameter was taken into account. Zou [30] and Cao [31] et al. analyzed the dynamic reliability of rubber with mixed uncertainties. Tsyss et al. [32] calculated the reliability of the rubber-metal damper using a computer simulation method. Czeslaw [33] studied the mechanics of sealing rings from the stress distribution via mechanical analysis, while the effect of the random distribution of material parameters on mechanical properties was ignored.

It can be concluded that the random parameters and the mission profile have a great influence on the reliability of rubber O-ring seals, and the mentioned literature provides insufficient evidence of the influence of randomness and application condition on the reliability. Therefore, this paper investigates the main failure modes and establishes the reliability model of the rubber O-ring seal on the basis of the analysis of the failure mechanism and mode. Moreover, the stochastic properties of the parameters were obtained through the designed experiments, and the unknown parameters under mission profile of the reliability model of O-ring were solved based on finite element analysis. Finally, the influences of parameter randomness on the reliability of O-rings were analyzed, and the sensitivity of parameter randomness to reliability was also obtained.

## 2. Reliability Model-Based Analysis of Rubber Materials under Application Condition

In the sealing structure, the main failure mode is leakage, caused by the failure of the rubber O-ring seal. The failure mechanism of the rubber O-ring seal based on the physical model is analyzed in this section, followed by reliability modeling. The randomness from the material, geometry, and load parameters of the rubber O-ring is discussed in detail to characterize the influences on reliability.

### 2.1. Failure Mechanism of Rubber O-Ring Seal

**Figure 1:**
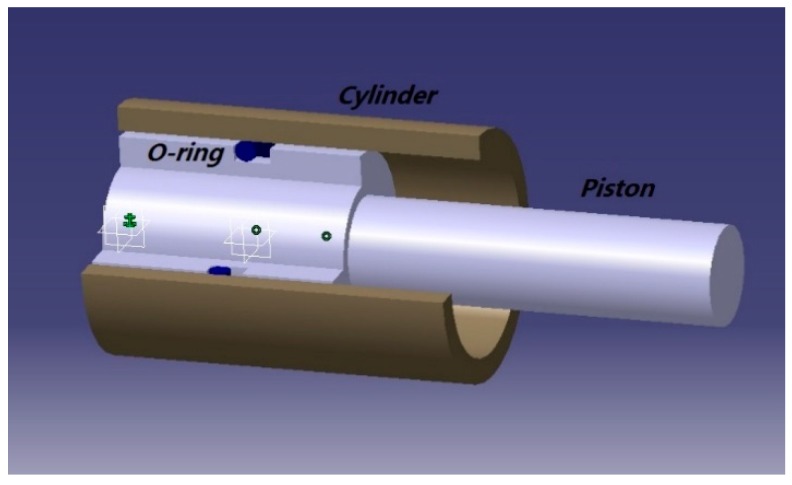
Geometry model of a rubber O-ring seal.

The sealing structure ensures that leakage does not occur by providing sealing stress on the O-ring, and part of the hydraulic system, including the rubber O-ring seal, is shown in Figure 1. Its reliability mainly depends on the sealing performance of the O-ring.

The stress concentration of the O-ring occurs in the compression process, and the inner diameter of the cylinder block may not be exactly the same due to errors caused in the machining process; in other words, the compression of the O-ring is more of a random variable than a constant. The uncertainty in compression may cause the maximum compressive stress of the rubber material $\sigma_{s}$ to exceed its ultimate strength $\sigma_{\max}$, which will lead to irreversible failure of the rubber material. As shown in Equation (1), when $k_{1}=1$, the rubber material is regarded to be in good condition without damage.

(1) $$k_{1}=\{\begin{array}{c} 0,\\ 1, \end{array}\begin{array}{c} \sigma_{\max}\leq \sigma_{s}\\ \sigma_{\max}>\sigma_{s} \end{array}.$$

Another possible failure occurs when the contact stress of the O-ring during compression is insufficient to prevent hydraulic oil leakage. According to the microscopic sealing principle [1,2], leakage occurs only when the oil pressure exceeds the contact stress. That is, the seal failure can be determined when the maximum contact stress $P_{c1}$ and $P_{c2}$ between the O-ring and the inner wall of the cylinder block and piston are less than the oil pressure $p_{oil}$. As shown in Equation (2), the sealing performance of the O-ring will be considered intact only when $k_{2}=1$, and no leakage will occur.

(2) $$k_{2}=\{\begin{array}{c} 0,\\ 1, \end{array}\begin{array}{c} other\\ P_{oil}<P_{c1}\;and\;P_{oil}<P_{c2} \end{array},$$

### 2.2. Reliability Modeling

The effects of compression on the maximum compressive stress of the rubber O-ring seal were analyzed based on the discussion in Section 2.1. Due to errors in machining, a change of compression will be caused by the unequal inner diameter of the cylinder block, while the maximum compressive stress of the O-ring $\sigma_{s}$ also changes, and the distribution of $\sigma_{s}$ is defined as $f\left(\sigma_{s}\right)$.

For maximum compressive stress $\sigma_{s}$ and limit stress $\sigma_{\max}$, it can be seen from Equation (1) that only when $\sigma_{s}<\sigma_{\max}$ is the O-ring regarded to be safe, and so the material reliability of the O-ring is shown in Equation (3):

(3) $$R_{s1}=\int_{0}^{\sigma_{\max}}f\left(\sigma_{s}\right)d\sigma_{s}.$$

Similarly, the change of load leads to the change of oil pressure during the movement of the piston, which can be seen in Equation (2); only when $P_{oil}<P_{\min}$ (where $P_{\min}=\min\left\{P_{c1},P_{c2}\right\}$) does the O-ring seal work well. The reliability of the O-ring seal is calculated as follows:

(4) $$R_{s2}=\int_{P_{oil}}^{+\infty }f\left(P_{\min}\right)dP_{\min},$$

among which oil pressure $P_{oil}$ is also a random variable.

Because the rubber O-ring seal only works normally when the material and seal are both reliable, the reliability model is established as Equation (5), which is obtained by synthesizing Equations (3) and (4).

(5) $$R_{s}=prob\left\{k_{1}=k_{2}=1\right\}=f\left(R_{s1},R_{s2}\right),$$

where $prob\left\{k_{1}=k_{2}=1\right\}$ represents the probability of occurrence of event $k_{1}=k_{2}=1$.

### 2.3. Random Characterization of Parameters on Reliability Model

The application of the rubber material is influenced by the randomness of the product, which usually comes from material, geometry, and load parameters. These parameters have comprehensive influences on the reliability of the rubber O-ring seal.

#### 2.3.1. Randomness of Material Parameters

The rubber O-ring seal is composed of a nonlinear hyperelastic material. In practical engineering (between 100% tension and 30% pressure), the Mooney–Rivlin [34,35,36] model is usually used to describe its material parameters, as shown in Equation (6):

(6) $$W=C_{10}\left(I_{1}-3\right)+C_{01}\left(I_{2}-3\right),$$

where $I_{1}$ and $I_{2}$ are two strain invariants and $C_{10}$ and $C_{01}$ are Mooney constants obtained using the stress–strain fitting method. Combining the relationship between stress and strain energy, the following equation can be obtained:

(7) $$\frac{\sigma_{1}}{2\left(\lambda_{1}^{2}-\frac{1}{\lambda_{1}}\right)}=C_{10}+\frac{1}{\lambda_{1}}C_{01},$$

where $\sigma_{1}$ and $\lambda_{1}$ are obtained from the test data. The test data can be fitted into a straight line, and then the values of material parameters $C_{10}$ and $C_{01}$ of rubber can be obtained. 

However, in the machining of the rubber O-ring seal, the values of material parameters $C_{10}$ and $C_{01}$ are not constant due to technological errors. This change of rubber material parameters has become a reflection of the randomness of material parameters in the reliability analysis of the rubber O-ring seal.

#### 2.3.2. Randomness of Geometric Parameters

According to the structural characteristics of the rubber O-ring seal in Figure 1, the cylinder and piston in the structure are usually machined by turning. In the process of machining, the inner diameter of cylinder $r_{1}$ and the outer diameter of piston $r_{2}$ will have certain errors—that is, the $r_{1}$ of each cylinder and the $r_{2}$ of the piston are not completely identical when they leave the factory, which leads to the random change of compression quantity of rubber O-ring seals. As shown in Equation (8), the compression quantity presents the randomness of normal distribution [3].

As Figure 1 shows, the cylinder and piston in the structure are usually machined by rotation. The inner diameter $r_{1}$ of the cylinder and the outer diameter $r_{2}$ of the piston will have certain errors—that is to say, the $r_{1}$ of each cylinder and the $r_{2}$ of each piston are not completely identical when they are released to market, which leads to random changes in the compression quantity of the rubber O-ring seal. The compression is a random variable subject to normal distribution, as Equation (8) shows:

(8) $$f\left(\Delta r\right)=\frac{1}{\sqrt{2\pi }\sigma_{\Delta r}}\exp\left[-\frac{{\left(\Delta r-\mu_{\Delta r}\right)}^{2}}{2\sigma_{\Delta r}^{2}}\right],$$

where Δr is the compression of the O-ring and $\mu_{\Delta r}$ and $\sigma_{\Delta r}$ are the mean and variance of O-ring compression, respectively.

#### 2.3.3. Randomness of Load Parameters

Similar to the randomness of the geometric parameters, the load of the rubber O-ring seal (i.e., oil pressure $P_{oil}$) is also a random variable. The oil pressure is related to the mission profile of the hydraulic system.

This paper considers the distribution of $P_{oil}$ during the whole life cycle of the rubber O-ring seal. In order to simplify the analysis, the oil pressure of the hydraulic system can be monitored by random sampling, and the statistical characteristics of $P_{oil}$ can be analyzed. Finally, the probability distribution of oil pressure is introduced into the reliability model to analyze the influence of $P_{oil}$ on the reliability of the rubber O-ring seal for further optimization.

## 3. Determination of Random Characterization of Parameters

Based on the established reliability model and the analysis of randomness in the materials, an aging test was conducted to obtain the degradation data of the rubber material as described in this section. Subsequently, the stochastic characteristics of the material, geometry, load, and stress were determined to analyze the reliability of the rubber O-ring seal.

### 3.1. Experimental Design for the Rubber Material

The aging data of rubber material parameters can be obtained by testing the rubber material’s properties under different aging experiments for the rubber O-ring. The aging data are stochastic.

**Figure 2:**
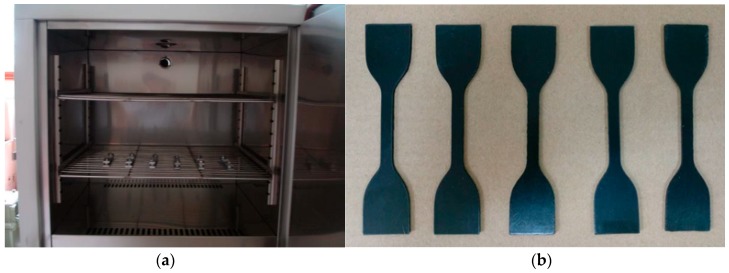
(**a**) Aging test chamber and (**b**) rubber samples.

In order to simulate the aging behaviors of different rubber samples under the working conditions, lubricant was applied to coat the surface of these samples, which were sealed in an aluminum-plastic bag with a special fixture. In addition, the rubber samples wrapped in aluminum-plastic were fixed in the test chamber to simulate the working temperature of the rubber samples in the working profile, as shown in Figure 2. In the experiment, the temperature of the environmental test chamber was set as 45 ± 5 °C, with a relative humidity of 50% ± 10%.

In this paper, a total of 10 groups of rubber material samples were selected for the aging test, and the rubber material samples were taken out 15 days later. A universal material testing machine was employed to conduct the tensile test for the aged rubber samples to obtain the stress–strain data, with which the stress–strain curves were obtained and then fitted into Equation (7), and the Mooney constants of rubber material samples at each time were obtained. The Mooney constants of these 10 groups of rubber samples were different in each test, and these Mooney constants determine the randomness of the material parameters of rubber samples at that moment. Based on the Mooney model, the change of the mechanical properties of rubber material is presented as the variation of $C_{10}$ and $C_{01}$, and the reliability is expressed as the function of $C_{10}$ and $C_{01}$. Therefore, the influences of the randomness in rubber material parameters on the reliability could be obtained with the combination of the aging test and the Mooney–Rivlin model.

Because the geometric parameters do not change with the working time, the randomness of the geometric parameters can be obtained by monitoring the distribution of the geometric parameters at one time. The original data of the O-ring seal were monitored to facilitate measurement. The existence of a lubricating medium causes errors in the measurement of geometric parameters. In this paper, we assumed that the sealing surface of the O-ring sealing structure was a standard circle ring. The geometric parameters of the sealing structure can be obtained by measuring the diameter of the ring of the sealing surface. Similarly, various diameter values can be measured from different seal structures, which determine the randomness of the geometric parameters of the seal structure.

The load on the rubber O-ring seal is influenced by oil pressure. The randomness of oil pressure determines the randomness of the load parameters. Therefore, the randomness of the load parameters on the rubber O-ring seal can be obtained by monitoring the change of oil pressure.

### 3.2. Random Parameters Analysis for the Rubber O-Ring Seal

Based on the test and monitoring method in Section 3.1, the material, geometric, and load parameters of the O-ring seal could be monitored, and the mean and variance of samples were calculated using Equations (9) and (10) (where $\mu =\bar{x}$) and then brought into Equation (11):

(9) $$\mu =\frac{1}{n}\sum_{i=1}^{n}x_{i},$$

(10) $$\sigma^{2}=\frac{1}{n}\sum_{i=1}^{n}{\left(x_{i}-\bar{x}\right)}^{2},$$

(11) $$y_{i}=\frac{x_{i}-\mu }{\sigma },i=1,2,\cdots ,n.$$

Here, we assumed that the variable $Y=\left(y_{1},y_{2},\ldots ,y_{n}\right)$ was subjected to a standard normal distribution, namely,Y∼N(0,1), then the statistic Z was constructed as shown in Equation (12) (where $\mu_{0}=0$, $\sigma_{0}=1$). A hypothesis test was performed on the distribution of the variable Y under the condition of a significance level α=0.05.

(12) $$Z=\frac{\bar{Y}-\mu_{0}}{\sigma_{0}/\sqrt{n}}$$

The data of the material parameters, geometric parameter, and load parameter were fitted according to Equations (9)–(12), and it was found that the material parameters $C_{10}$ and $C_{01}$, the geometric parameter Δr, and the load parameter $P_{oil}$ all obeyed normal distributions. The obtained results are shown in Table 1.

### 3.3. Random Characterization of Stress under Actual Application Conditons

**Figure 3:**
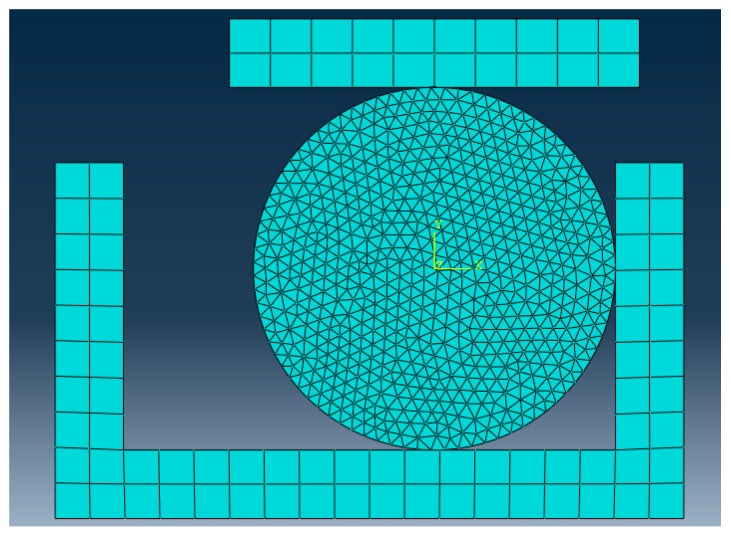
Finite element model of the O-ring.

A finite element model was established in ABAQUS to analyze the stress in the O-ring, as shown in Figure 3. In the model, the CPE4R grid was selected for the sealing structure component and the CPS3 grid was chosen for the rubber component. A total of 1233 units were divided in the rubber component.

**Figure 4:**
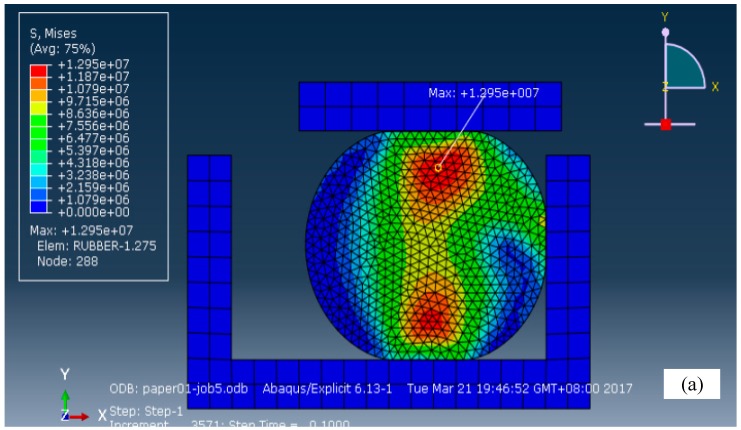

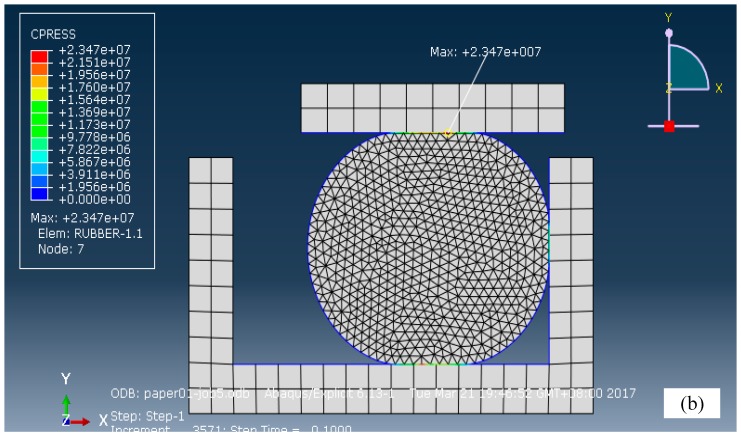
(**a**) Compressive stress and (**b**) contact stress distribution nephograms.

In addition, the four parameters in Table 1 were selected as their mean values to define the relevant parameters in the preprocessing for the submission of calculations. The results are shown in Figure 4. Figure 4a shows the compressive stress distribution nephogram of the rubber O-ring seal. From the figure, it can be seen that the stress distribution was of dumbbell-type, and the maximum value of compressive stress $\sigma_{s}$ = 12.95 MPa, the ultimate stress of rubber material $\sigma_{m}$ = 20 MPa (i.e., $\sigma_{s}<\sigma_{m}$). Therefore, the rubber material will not fail due to the stress concentration at this moment. Figure 4b shows the contact force distribution nephogram of the rubber O-ring seal. The maximum contact stress between the O-ring seal and the cylinder wall and the O-ring seal and the piston was 23.47 MPa and 21.89 MPa, respectively. The oil pressure $P_{oil}$ = 10 MPa; both $P_{c1}$ and $P_{c2}$ were greater than $P_{oil}$. Hence, the sealing performance of the O-ring seal was considered to be intact.

Owing to the randomness of material, geometric, and load parameters, the stress distribution of the rubber O-ring seal also showed stochastic characteristics. However, it is difficult for the finite element software to show these random factors in the pretreatment process. A sampling method was employed to gain the distribution of the working stress.

**Figure 5:**
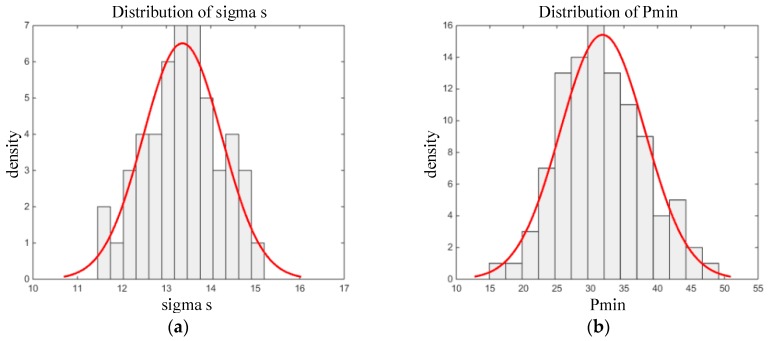
Distribution of (**a**) compressive stress and (**b**) contact stress.

Therefore, the geometric parameters Δr in Table 1 were discretized by MATLAB to generate 50 sets of sample data, and the finite element software ABAQUS was invoked cyclically to calculate the maximum compressive stress $\sigma_{s}$ of the O-ring and the smaller contact stress ($P_{\min}=\min\left\{P_{c1},P_{c2}\right\}$) between $P_{c1}$ and $P_{c2}$ of the two sealing faces. The results are shown in Figure 5. The results show that both $\sigma_{s}$ and $P_{\min}$ followed normal distribution.

### 3.4. Reliability Analysis

Based on the results of the simulation analysis, we can see that $\sigma_{s}$ was in accord with a normal distribution with mean $\mu_{s}$ = 13.3627 and variance $\sigma_{s}^{2}$ = 0.78001. Based on Equation (3), the reliability was calculated as:

(13) $$\begin{array}{cc} R_{s1} & =\int_{0}^{\sigma_{\max}}\frac{1}{\sqrt{2\pi }\sigma }\exp\left[-\frac{{\left(\sigma_{s}-\mu \right)}^{2}}{2\sigma^{2}}\right]d\sigma_{s}=\int_{-\frac{\mu }{\sigma }}^{\frac{\sigma_{\max}-\mu }{\sigma }}\frac{1}{\sqrt{2\pi }}\exp\left(-\frac{x^{2}}{2}\right)dx\\ & =\Phi \left(\frac{\sigma_{\max}-\mu }{\sigma }\right)-\Phi \left(-\frac{\mu }{\sigma }\right)=\Phi \left(7.5152\right)-\Phi \left(-15.1302\right)\approx 1 \end{array}$$

where Φ(x) is the cumulative distribution function of the standard normal distribution.

Similarly, $P_{\min}$ also displayed a normal distribution, whose mean and variance were $\mu_{p}$ = 31.8179 and $\sigma_{p}^{2}$ = 39.5939, respectively. Based on the distribution of $P_{oil}$ in Table 1, according to Equation (4), the reliability was solved as:

(14) $$\begin{array}{cc} R_{s2} & =\int_{-\frac{\mu_{p}-\mu_{o}}{\sqrt{\sigma_{p}^{2}+\sigma_{o}^{2}}}}^{+\infty }\frac{1}{\sqrt{2\pi }}\exp\left(-\frac{y^{2}}{2}\right)dy=\Phi \left(\frac{\mu_{p}-\mu_{o}}{\sqrt{\sigma_{p}^{2}+\sigma_{o}^{2}}}\right)\\ & =\Phi \left(\frac{31.8179-10}{\sqrt{39.5939+4}}\right)=0.999524 \end{array}$$

At this moment, the material reliability and sealing reliability of the rubber O-ring seal could be obtained. According to Equation (5), the reliability of the rubber O-ring seal material and sealing reliability intersected as shown in Equation (15). The reliability of the rubber O-ring seal seals under Δr random condition was calculated as:

(15) $$R_{s}=f\left(R_{s1},R_{s2}\right)=0.9995.$$

## 4. Influence of Random Parameters and Discussion

In practical production, the quality of a product is usually represented by a control chart, and the control limit is determined according to the 6σ principle. At this time, the reliability level of the product was 99.73% [37]. According to the analysis in Section 3.4, the reliability of the O-ring was sufficient to meet the requirements of product quality in the production process. Therefore, this section focuses on analyzing the influence of the randomness of the parameters that affect the reliability of O-ring. 

### 4.1. Influence of Material Parameters

**Figure 6:**
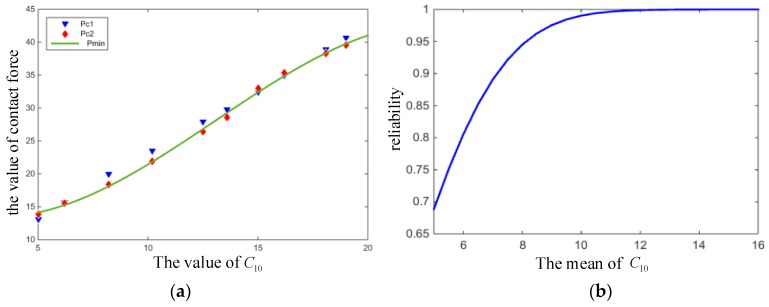
(**a**) Contact stress and (**b**) reliability with varying $C_{10}$.

**Figure 7:**
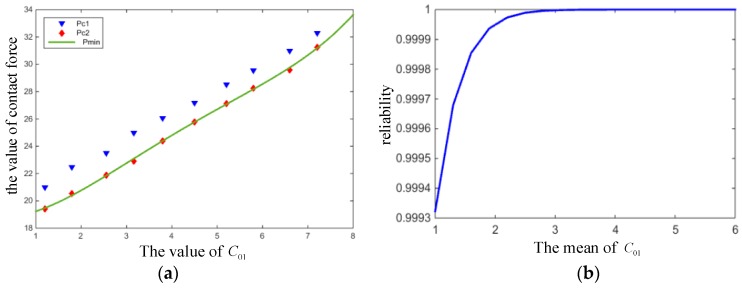
(**a**) Contact stress and (**b**) reliability with varying $C_{01}$.

The variation of rubber material parameters $C_{10}$ and $C_{01}$ affect the contact force of the O-ring. Figure 6 and Figure 7 show the variation of the maximum contact stress of the O-ring and the reliability of the O-ring with $C_{10}$ and $C_{01}$, respectively. It can be seen from the two figures that with the increase of $C_{10}$ and $C_{01}$, the contact force also raised continuously, and that the greater the values of $C_{10}$ and $C_{01}$, the higher the sealing reliability of the O-ring.

### 4.2. Influence of Geometric Parameters

**Figure 8:**
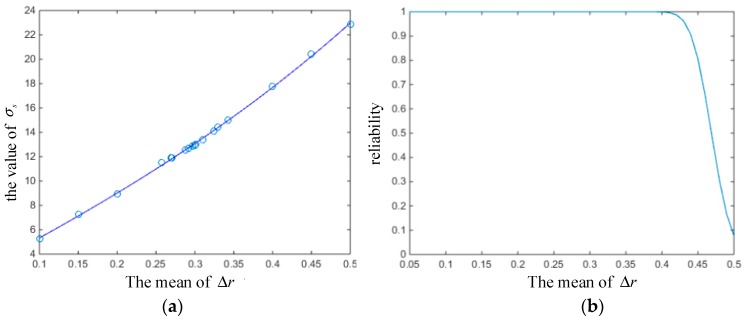
(**a**) Compressive stress and (**b**) reliability with varying compression quantity Δr.

Process limitations lead to the change of compression Δr. Figure 8 demonstrates the variation curves of maximum compression stress $\sigma_{s}$ and the reliability of the O-ring with the mean of compression Δr, respectively. It can be seen from the graphs that $\sigma_{s}$ increased constantly with the increase of Δr. Moreover, the reliability curve shows that minor compression had little influence on reliability, but reliability decreased sharply when the mean value of Δr was greater than 0.4. 

### 4.3. Influence of Load Parameters

**Figure 9:**
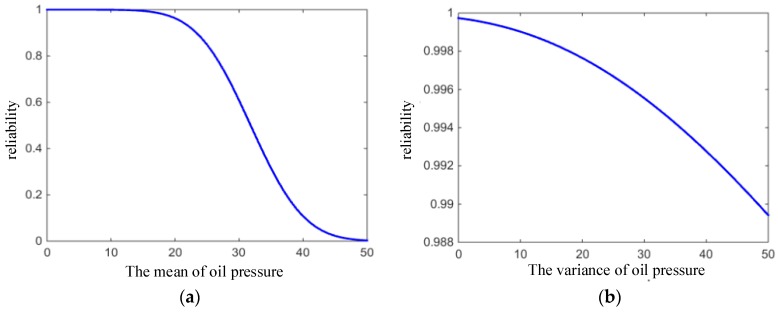
Reliability varying with the (**a**) mean and (**b**) variance of oil pressure.

For rubber O-ring seals, the operating conditions directly affect the change of oil pressure. Figure 9 shows the variation of reliability with the mean and variance of oil pressure change. When the mean of $P_{oil}$ was greater than 15, the reliability began to decrease, but when the variance of $P_{oil}$ increased from 0 to 50, the reliability only decreased from 0.9997 to 0.9894. It can be concluded that the mean of $P_{oil}$ has a greater influence on the reliability of O-rings than that of variance.

## 5. Conclusions

This paper began with the parameters affecting the reliability of the O-ring seal, considering the influence of randomness on the performance of the O-ring. From the results of case studies, the following conclusions can be drawn:

- Defects in processing technology lead to changes in the compression of the O-ring. With the increase of compression, compression stress increases, which greatly affects the reliability of the O-ring when compression reaches a certain degree.
- When the Mooney–Rivlin constitutive model constants of rubber material, $C_{10}$ and $C_{01}$, change with ageing, the maximum contact stress of the O-ring also changes. It can be seen from the comparison that the larger $C_{10}$ and $C_{01}$ are, the greater the maximum contact stress of the O-ring seal, and the better the sealing performance of the O-ring.
- Random change of load leads to random change in oil pressure, which has a great influence on the reliability of the O-ring seal. The input of the load should be optimized, and the influence of an appropriate shunt load on the O-ring should be considered.
- This method of analyzing the influence of parameter changes on the reliability of the O-ring seal can reduce field test time and improve the economy and timeliness of the O-ring seal.

## Figures and Tables

**Table 1:** Randomness of parameters in the rubber O-ring seal.

Parameter	Distribution Type	Mean	Variance
$C_{10}$	Normal	12.5 Mpa	6.25
$C_{01}$	Normal	4.2 Mpa	1
Δr	Normal	0.3 mm	0.002
$P_{oil}$	Normal	10 Mpa	4
